# Varicocele

**Published:** 1890-09

**Authors:** 


### VARICOCELE.

A patient, aged thirty-two, a jeweler by occupation, had had a varicocele on the left side of the scrotum for a period of about eight years. As a result of this condition the left testicle became atrophied to about half its normal size, and there was a marked elongation of the scrotum, with considerable pain.

It may be of interest here to note that varicocele usually occupies the left side, and the reason given by Prof. Wyeth was that the left vein, owing to its long course from the testicle to its termination in the left renal vein, has a heavier column of blood to support, and that it enters at right angles to that vein and to its blood-current. Again the left testicle is heavier than the right. Mr. Curling has stated that cases of varicocele are often associated with varicose veins of the lower extremities or elsewhere.

In the cases that had been examined by Dr. Wyeth the combination referred to was very rarely found to exist, and in those cases in which it did exist the varicocele was generally very large. It is to be noted, too, that varicose veins of the extremities are found usually in either middle life or among the aged, whereas varicocele is generally met with soon after the advent of puberty. Varicocele is most prevalent between the ages of fifteen and thirty-five—the period of the greatest activity of the sexual organs, when they demand for the exercise of their function a considerable supply of blood.

Varicocele is a very common disease, but usually passes unnoticed, as it usually gives rise to no pain. The veins enlarge slowly. Eventually there is a sense of weight and uneasiness in the testicle and cord of the affected side. This condition is aggravated by standing for any length of time, or remaining in a constrained position, and by violent muscular exercise.

On stripping the patient a flaccid condition of the scrotum is noticed, and the testicle of the affected side hangs down much lower than it ought to do. The tumor is soft and elastic to the touch, and the dilated veins are readily seen and felt. They can be rolled under the skin, and from their feel are usually compared to a bag of worms. On asking the patient to cough the dilatation of the veins gives rise to an impulse which is gentle and not forcible like that communicated by a hernia. The swelling of an inguinal hernia is spherical and resonant on percussion, when composed of intestine. If the hernia is reducible, and is returned into the abdominal cavity with the patient in the recumbent position, and if the index finger be carried into the internal ring and held there while the patient is made to assume the erect posture, the hernia will be prevented from descending, while the veins will again refill and demonstrate the existence of a varicocele.

Having determined the existence of a varicocele in this man's case, an operation was done at the Polyclinic for the relief of this condition. After the scrotum and pubes had been thoroughly shaved and washed with ether and sublimate solution, an incision was made over the cord from the external ring down to the testicle, carefully separating the vas deferens and the spermatic artery. The veins were tied close to the external ring above with catgut ligatures, and were then carefully dissected out below that point as far down as the testicle of the affected side, where they were again tied. Catgut sutures were next carried through the stub of veins above and below, and tied up short, holding the testicle well up to the external ring. The scrotum was then amputated by the use of King's clamp. The wound was sewn up by the continuous silk suture, no drainage tube being applied. Healing took place by first intention.

The patient was discharged, cured sixteen days after the operation.—*International Journal of Surgery.*

